# Editorial: Updates on RIG-I-like receptor-mediated innate immune responses

**DOI:** 10.3389/fimmu.2023.1153410

**Published:** 2023-02-08

**Authors:** Uday Kishore, Thomas A. Kufer

**Affiliations:** ^1^ Department of Veterinary Medicine, United Arab Emirates University, Al Ain, United Arab Emirates; ^2^ Medical Research Council Immunochemistry Unit, Department of Biochemistry, University of Oxford, Oxford, United Kingdom; ^3^ Department of Immunology, Institute for Nutritional Medicine, University of Hohenheim, Stuttgart, Germany

**Keywords:** innate immunity, PAMP (pathogen-associated molecular pattern), immune escape, RIG1 receptors, signalling, transcription factor

Innate immunity is designed to recognise conserved microbial structures, called pathogen-associated molecular patterns (PAMPs), by germline-encoded receptors in the host, referred to as pattern-recognition receptors (PRRs) (1). In mammals, PAMP-PRR engagement is important for controlling bacterial and viral infections for potentiating innate immune clearance as well as for inducing adaptive immune responses. Many pathogens, especially viruses, can invade the host target cells, and hence, the sensing of foreign nucleic acids in the cytosol of host cells, in particular RNA derived from evading viruses, is a pivotal part of the anti-viral innate immune response. The two cytosolic helicases RIG-I (retinoic acid-inducible gene-I; DDX58) and MDA5 (melanoma-differentiation-associated gene 5), commonly referred to as RLRs (RIG-I like helicases) act as sensors for foreign (non-self) nucleic acids. A wealth of work identified the structural motifs necessary for the activation of these innate immune sensors and their signalling pathways that involve interaction with the mitochondrial protein MAVS for downstream activation of type-I interferon responses [reviewed in (2)]. The importance of a rapid type-I interferon response to control viral infection is nicely illustrated by genetic predispositions for low type-I interferon responses and levels in human that correlate with fatal outcomes to coronavirus (SARS-CoV-2) infection (3).

This Research Topic summarizes novel aspects of the molecular regulation of RLR activation and signalling and the subversion of this pathway by viral pathogens.

In their article, Kouwaki et al. show that both MDA5 and RIG-I are involved in sensing of SARS-CoV-2 viral RNA. Moreover, their work confirms recent data and reveals that SARS-CoV-2 virus can actively dampen this recognition by inhibiting RIG-I interaction with MAVS and destabilization of TBK1, mediated by the viral proteins 9a and 7a, respectively. In this context, it is worth noting that adaptation of the immune response towards a given pathogen is essential; inappropriate or exaggerated immune responses can have detrimental outcomes for the host. Insufficient tolerance towards viral infections contributes to most viral disease symptoms and is the cause of most fatal outcomes of viral infections. Using transmissible gastroenteritis virus (TGEV), a relevant pathogen of livestock, Pu et al. show all-trans retinoic acid (ATRA) can attenuate inflammatory responses towards TGEV, partly by inhibiting virus-induced expression of PRRs, including RLRs.

An important issue in the RLR signalling pathway and type-I interferon induction is ubiquitylation of cellular signalling proteins. RIG-I activation, for example, is dependent on ubiquitylation; more than 15 years ago, TRIM25 was identified as a critical factor for licencing RIG-I activation (4). Three articles in this special issue focus on ubiquitylation in viral sensing. Oshiumi briefly summarizes our current understanding of ubiquitin ligases in the regulation of RIG-I- and MDA5-mediated antiviral response, focussing on both the E3 ligases that mediate K63-liked ubiquitylation for regulatory events as well as on those E3 ligases that induce K48-linked ubiquitin-mediated degradation. This is accompanied by a comprehensive review by Huang et al. that summarizes how viruses have evolved to target these pathways to escape innate immune sensing. The complex subversion mechanisms used by coronaviridae to evade type-I interferon induction is further exemplified by the work of Ran et al., who have provided experimental data to explain how the papain-like protease of SARS-CoV-2 (SCoV2-PLpro) interferes with anti-viral innate immune sensing. The study provides evidence that SCoV2-PLpro protease inhibits anti-viral innate immunity by targeting RIG-I and components of the downstream signalling pathway, which is independent of the main deubiquitinase activity of the enzyme.

Using *Miichthys miiuy*, a telost fish and derived cell lines, Yan et al. show that the RIG-I signalling adaptor mediator of IRF3 activation (MITA), an adaptor in the RLR signalling, is targeted for ubiquitin-mediated proteasomal degradation by IRF4b or IRF8 *via* their core domain. IRF4b and IRF8 also have an inhibitory function on MITA-mediated NF-κB signaling pathway. The study provides useful insight into regulatory mechanisms in fish innate immune system.

Since the first description of RIG-I and MDA5 as RNA sensors (5, 6), we now have a reasonable understanding of their molecular functions and signalling. The finding that RIG-I is also involved in a pathway for cytosolic DNA sensing (7, 8) seems to suggest a central role for RIG-I in innate immune response towards cytosolic nucleic acids in general. However, our understanding of the complex regulatory mechanisms involving these important PRRs and their co-evolution with viruses and their adaptation to subvert viral activation, is still incomplete. The collection of research papers in this special issue adds to our understanding of the function of these fascinating proteins. The review articles provide a great start point for non-experts to get them excited about the field in addition to being an excellent resource for experts and established scientists.

## Author contributions

TK wrote the first draft; UK edited and revised the manuscript. Both TK and UK read and approved the final version.

## References

[1] Brubaker SW Bonham KS Zanoni I Kagan JC. Innate immune pattern recognition: A cell biological perspective. Annu Rev Immunol (2015) 33:257–90. doi: 10.1146/annurev-immunol-032414-112240 25581309 PMC5146691

[2] Roers A Hiller B Hornung V. Recognition of endogenous nucleic acids by the innate immune system. Immunity (2016) 44(4):739–54. doi: 10.1016/j.immuni.2016.04.002 27096317

[3] Zhang Q Bastard P Effort CHG Cobat A Casanova JL. Human genetic and immunological determinants of critical COVID-19 pneumonia. Nature (2022) 603(7902):587–98. doi: 10.1038/s41586-022-04447-0 PMC8957595 35090163

[4] Gack MU Shin YC Joo CH Urano T Liang C Sun L. TRIM25 RING-finger E3 ubiquitin ligase is essential for RIG-i-mediated antiviral activity. Nature (2007) 446(7138):916–20. doi: 10.1038/nature05732 17392790

[5] Yoneyama M Kikuchi M Matsumoto K Imaizumi T Miyagishi M Taira K. Shared and unique functions of the DExD/H-box helicases RIG-I, MDA5, and LGP2 in antiviral innate immunity. J Immunol (2005) 175(5):2851–8. doi: 10.4049/jimmunol.175.5.2851 16116171

[6] Yoneyama M Kikuchi M Natsukawa T Shinobu N Imaizumi T Miyagishi M. The RNA helicase RIG-I has an essential function in double-stranded RNA-induced innate antiviral responses. Nat Immunol (2004) 5(7):730–7. doi: 10.1038/ni1087 15208624

[7] Ablasser A Bauernfeind F Hartmann G Latz E Fitzgerald KA Hornung V. RIG-i-dependent sensing of poly(dA:dT) through the induction of an RNA polymerase III-transcribed RNA intermediate. Nat Immunol (2009) 10(10):1065–72. doi: 10.1038/ni.1779 PMC3878616 19609254

[8] Chiu YH Macmillan JB Chen ZJ. RNA Polymerase III detects cytosolic DNA and induces type I interferons through the RIG-I pathway. Cell (2009) 138(3):576–91. doi: 10.1016/j.cell.2009.06.015 PMC2747301 19631370

